# Brain Injury: How Dietary Patterns Impact Long-Term Outcomes

**DOI:** 10.1007/s40141-023-00413-7

**Published:** 2023-07-05

**Authors:** Palak R. Patel, Patrick Armistead-Jehle, Nicholas R. Eltman, Kelly M. Heath, David X. Cifu, Randel L. Swanson

**Affiliations:** 1 Department of Physical Medicine and Rehabilitation, University of Pennsylvania Perelman School of Medicine, Philadelphia, PA USA; 2 Munson Army Health Center, Concussion Clinic, Fort Leavenworth, KS USA; 3 Corporal Michael J. Crescenz VA Medical Center, Center for Neurotrauma, Neurodegeneration, and Restoration, Philadelphia, PA USA; 4 Rowan-Virtua School of Osteopathic Medicine, Stratford, NJ USA; 5 Rehab Medicine Service, Corporal Michael J. Crescenz VA Medical Center, 3900 Woodland Avenue, Mail Stop #117, Philadelphia, PA 19104 USA; 6 Physical Medicine and Rehabilitation, Virginia Commonwealth University School of Medicine, Richmond, USA; 7 Physical Medicine and Rehabilitation, Central Virginia Veterans Health Care System, Richmond, USA

**Keywords:** Traumatic brain injury, Concussion, Nutrition, Diet, Lifestyle interventions

## Abstract

**Purpose of Review:**

Individuals with a history of traumatic brain injury (TBI) are at a much greater risk for developing cardiovascular disease (CVD) compared to the general population. This review discusses dietary patterns as a means of addressing modifiable risk factors following TBI exposure. Evidence-based resources for practicing Physiatrists and Brain Injury Medicine specialists pertaining to nutrition education and counseling are also provided.

**Recent Findings:**

We examined Mediterranean, Dietary Approaches to Stop Hypertension, plant-based, ketogenic, and intermittent fasting dietary patterns through publications of clinical trials and systematic reviews. While many reviews had significant positive findings, some were limited by generalizability.

**Summary:**

While there is extensive literature on the immediate nutrition goals in the inpatient setting following an acute TBI exposure, there is limited literature discussing the nature of diet and nutrition in the post-acute setting. Fortunately, most individuals with TBI exposure survive their initial injury and continue into the recovery phase. The scientific literature supports increased morbidity and mortality with chronic TBI exposure compared to matched counterparts, most notably with CVD. A diet rich in fiber and nutrients but limited in added sugars, saturated fats, and excess calories would likely have the greatest cardiovascular and related neurologic protection. Future studies are needed to assess the specific impact of dietary interventions in the chronic phase of brain injury recovery.

## Introduction

According to the CDC, there were more than 220,000 hospitalizations and 69,000 deaths related to a traumatic brain injury (TBI) in the USA in 2021. These numbers significantly underestimate the actual affected population, as they do not incorporate those who present for care in the emergency department, primary care offices, or urgent care centers, and those who did not seek medical care [1]. Gender differences also exist, with males being twice as likely to be hospitalized and three times as likely to die than females, with these differences lessening over the age of 65 [2, 3]. While the prevention of TBI is a crucial initiative, it is also important to note that many of these individuals survive their initial TBI exposure. It is estimated that about 1.1% of the US population has had TBI exposure [3]. As a result, many individuals enter the recovery phase of TBI, which also presents unique challenges and required adaptations. This review summarizes the literature on individuals in the chronic phase of TBI recovery, the causes of morbidity and mortality within this cohort, and strategies to help mitigate modifiable risk factors through dietary means. Due to the volume of literature on specific dietary patterns and their effect on overall health, well-conducted systematic reviews and meta-analyses are used as primary references for this narrative review.

## Long-Term Outcomes of TBI Exposure

TBI exposures are more frequent in young males and adults over the age of 65, most often due to falls, motor vehicle accidents, blunt-force trauma, violence, and self-harm [3]. From 2001 to 2010, it is estimated that around 2.5 million people presented to the emergency department following a TBI exposure annually, and of those, 220,000 were hospitalized, and about 50,000 people died [2]. The TBI mortality rate is about 2% of those who seek care; thus, most individuals survive their initial injuries. Evaluating the modifiable factors that contribute to cognitive function and long-term outcomes can equip healthcare professionals to provide the best support to TBI survivors throughout the lifespan following TBI exposure.

Using data obtained from the TBI Model Systems (TBIMS) National Database (NDB) cohort, one study described the life expectancy following TBI exposure in individuals who transitioned to an inpatient rehabilitation facility (IRF) from their initial acute care hospitalization [4]. Nearly 7000 individuals were included and evaluated in acute care and IRF, and then at 1 year, 2 years, 5 years, and subsequent 5-year increments. Standardized mortality ratios (SMR) were calculated and compared to the matched general population. Life expectancy after TBI exposure was reduced by about 9 years. Relative to age, gender, and race-matched controls, those with a history of TBI exposure had a 50-fold increased risk of death by seizure, a tenfold increased risk of accidental poisoning, a fivefold increased risk by fall or homicide, and a twofold increased risk of suicide or mental health conditions. However, the most significant cause of mortality was cardiovascular disease (CVD), contributing to 26% of the deaths in those with a history of TBI (SMR of 1.42) [4].

A prospective cohort study followed over 4300 individuals with TBI exposure and no other significant medical comorbidities for 10 years [5••]. Compared to their age, gender, and race-controlled counterparts, those with mild or moderate TBI severity had greater risks of developing cardiovascular, endocrine, neurologic, or psychiatric conditions that, in turn, were linked with higher mortality rates. Following injury, researchers found hazard ratios (HR) to be 1.34 for hypertension, 2.2 for coronary artery disease, 6.2 for adrenal insufficiency, 3.8 for substance misuse, and 3.0 for dementia. These risks were also demonstrated at an earlier age than that of their matched counterparts, most notably in the 18- to 40-year-old age group. Importantly, this study differed from prior research in that it specifically revealed a connection between TBI exposure and the development of circulatory disease in all age cohorts regardless of past medical history [6]. The authors noted that the specific causes of increased comorbidity rates are not well understood and are likely secondary to multiple factors including injury cascade alterations in hormonal and inflammatory balances, other injuries associated with the initial TBI exposure, and overall lifestyle changes regarding physical activity and diet, with associated downstream metabolic changes [5••].

## Cardiovascular Disease Risk Following TBI Exposure

Literature on the brain–heart connection dates back to the 1950s, with research demonstrating the negative impact of sympathetic nervous system hyperactivity on several cardiac conditions, like arrhythmias and cardiomyopathies [7]. However, few studies have evaluated the brain–heart connection as a function of TBI. A large, retrospective cohort study investigated the development of CVD following TBI in over 1.5 million post-9/11-era veterans [8]. In line with previous studies, the authors found that relative to veterans without a history of TBI exposure, those with a TBI exposure history had a higher risk of CVD. Moreover, CVD risk varied as a function of TBI severity, with a CVD HR of 1.62 for mild TBI exposure, HR of 2.63 for moderate-to-severe TBI exposure, and HR of 4.60 for penetrating TBI exposure. Study participants were predominantly young males, which again supports the concept that TBI contributes to an accelerated risk of CVD and related end-organ pathologies with increased age [8].

Recently, work has been done to explore the brain–heart connection from a neuropathological perspective. A recent review summarized the available pre-clinical and clinical literature investigating both traumatic and non-traumatic cerebral microvasculature pathology (CMP) and hypothesized that CMP is a common pathophysiological consequence of both TBI exposure and CVD, each of which gives rise to accelerated age-related cognitive decline and Alzheimer’s disease or related dementias [9]. TBI exposure results in acute mechanical disruption of the blood–brain barrier (BBB), allowing indiscriminate extravasation of blood components into the brain parenchyma, often with downstream pathophysiological consequences [10–13]. In contrast, the presence of a poor modifiable cardiovascular risk factor profile — from a combination of conditions including hyperglycemia, dyslipidemia, hypertension, obesity, tobacco use, and physical inactivity — generates daily microtrauma to the cerebral microvasculature along with the promotion of atherosclerosis and arteriosclerosis, which cause non-traumatic BBB disruption with accompanying extravasation of blood components into the brain parenchyma. Consistent with this hypothesis, a large retrospective cohort study in over 180,000 veterans demonstrated a significant additive association between TBI exposure and CVD on dementia risk, supporting the clinical recommendation for secondary cardiovascular risk factor reduction post-TBI exposure, including a healthy diet and other lifestyle interventions [9, 14].

It is also important to consider the chronic effects of TBI exposure, and its overall impact on morbidity and mortality. As suggested above, many individuals are surviving their initial TBI exposure. However, TBI survivors are found to be at an increased risk of developing common comorbidities when compared to the general population [4, 5••, 6]. These studies suggest that relative to the general population, individuals with a history of TBI exposure have a higher risk of significant morbidity and mortality related to CVD and other metabolic disorders. Therefore, it is crucial to emphasize cardiovascular health following TBI exposure and to focus on the reduction of modifiable cardiovascular risk factors for secondary risk reduction following neurotrauma. These can often be addressed through lifestyle interventions, which include changing dietary habits. Below we review specific dietary patterns (overview provided in Table 1), and current research on their connection with CVD and brain health.

**Table 1:** Overview of dietary patterns

	Mediterranean diet (MD)	Dietary Approaches to Stop Hypertension (DASH)	Plant-based diets (PBD)	Ketogenic diet (KD)*	Intermittent fasting (IF)
Foundation of dietary pattern	Whole plant–based foods, including whole grains, vegetables, fruits, legumes, nuts, seeds, herbs, spices, and olive oil + foods rich in omega-3 fatty acids or with high monounsaturated/saturated fat ratio	Whole plant–based foods, including whole grains, vegetables, fruits, legumes, nuts, seeds, herbs, and spices + Sodium intake limited to < 2300 mg per day	Whole plant–based foods, including whole grains, vegetables, fruits, legumes, nuts, seeds, herbs, and spices + Avoid all animal-based foods (if vegan), or all animal-based foods except dairy and eggs (if vegetarian)	Consumption of high-fat foods, along with moderate protein and very low carbohydrate. Goal is to achieve ketosis by limiting carbohydrate intake, thereby preventing the body from relying on glucose as primary fuel source and instead utilizing ketones	Time-restricted eating (as opposed to ad libitum eating). Multiple variations of time restriction are utilized, including fasting for one or more days per week, or fasting for a set number of hours each day
Dietary allowances	Fish, seafood, dairy, and poultry in moderation	Fish, seafood, poultry, and low-fat dairy in moderation	All whole-food-based non-animal products	High-fat foods	All major food groups/types generally allowed
Recommended dietary limitations	Red meat, sweets	Full-fat dairy, red meat, sugary processed foods	Plant-based substitutions for animal-based foods	N/A	N/A
Dietary restrictions	Highly/ultra processed foods	Highly/ultra-processed foods; sodium to < 2300 mg per day	Highly/ultra-processed foods; animal-based foods	Carbohydrates, especially highly/ultra-processed carbohydrates	Time (varies); highly/ultra-processed foods

^*^Not recommended for long-term use (> 6 months) without medical supervision and monitoring

## Dietary Patterns

### Mediterranean Diet

Broadly defined, a Mediterranean diet (MD) consists of whole plant–based foods, including fruits, vegetables, legumes, nuts, seeds, herbs, spices, olive oil, and whole grains. Fish (especially those high in omega-3 fatty acids), other seafood, dairy, and poultry are included in moderation, while red meats and sweets are consumed infrequently. Highly or ultra-processed foods are absent. Interest in this diet was piqued in the 1950s when it was observed that relative to the US and Northern European countries, heart disease was much less prevalent in countries that bordered the Mediterranean Sea [15].

Several studies have evaluated the effect of the MD on aspects of metabolic health [16, 17]. A systematic review and meta-analysis of randomized controlled trials in adults looked at the effects of the MD compared to no treatment, usual care, or different diets on metabolic syndrome incidence, components, and risk factors [18•]. Ultimately, 84 papers, representing 57 unique clinical trials, were identified. Analyses indicated that 18 of 28 studies included assessments of metabolic syndrome components, and risk factors improved with the MD, including body weight, BMI, BP, glucose, insulin, lipids, and markers of inflammation. Moreover, the MD was associated with a lower risk of CVD and stroke (risk ratios of 0.61 and 0.67, respectively) [18•]. A scoping review evaluated 36 clinical and observational studies published between 2011 and 2021 that evaluated the influence of a MD on atherosclerosis and associated risk factors in subjects with and without diabetes [19]. The authors concluded that a MD is related to improved biomarkers, atherosclerotic plaque, and anthropometric measurements linked to CVD. However, they noted that most clinical studies were done in Mediterranean countries and most observational studies were cross-sectional with limited long-term follow-up, potentially limiting the generalizability of study findings [19].

Beyond general metabolic health, studies have evaluated the influence of the MD on cognitive health [20, 21]. One study evaluated MD adherence and the risk of incident stroke in a prospective, population cohort of over 23,000 UK residents [22]. Across the 17-year follow-up period, stroke risk was significantly lower in those following a MD more strictly based on their dietary assessments compared to those who were less adherent (HR = 0.83, *p* < 0.01). Stroke risk was also reduced among those at high risk of CVD, as measured by the Framingham risk score. However, the authors noted that the positive findings were largely driven by females, but were unable to uncover explanatory factors due to the low number of stroke incidence in their study population [22]. Another randomized, controlled trial evaluated cardiovascular disease risk as a function of low-fat/high complex carbohydrate diet versus a MD, recording adherence with a validated questionnaire [23]. After 5 years, those in the MD group had decreased plaque size and atherosclerosis progression, as measured by intima-media thickness and plaque within the carotid arteries, relative to baseline, while those in the low-fat diet group did not. These authors concluded that a MD has clinical utility in the context of secondary CVD prevention [23]. In another meta-analysis, researchers evaluated stroke risk as a function of MD in over 680,000 individuals from 20 cohort studies [24]. A lower risk of both ischemic and hemorrhagic stroke was associated with better adherence to the MD in both Mediterranean and non-Mediterranean cohorts (relative risks ranging from 0.76 to 0.86) [24]. Lastly, a 2019 Cochrane Review evaluated 30 randomized controlled trials (RCTs) to identify the influence of a Mediterranean-style diet on primary and secondary prevention of cerebral vascular disease [25]. The reviewers defined a Mediterranean-style diet as having a high monounsaturated/saturated fat ratio, high intake of plant-based foods, low-to-moderate red wine consumption, high consumption of whole grains and cereals, low consumption of meat and increased consumption of fish, and moderate consumption of dairy. The review concluded that few trials reported the occurrence of CVD. Of those that did, there was low to moderate quality data indicating that a MD was associated with decreased rates of stroke and death from a heart attack or other causes. Some other studies measured risk factors for CVD, with low to moderate quality evidence for some beneficial changes to lipid levels and blood pressure. The authors ultimately concluded that there remains uncertainty regarding the effects of a Mediterranean-style diet on the primary and secondary prevention of CVD [25].

### DASH Diet

Dietary Approaches to Stop Hypertension (DASH) was coined over two decades ago by the National Heart, Lung, and Blood Institute of the NIH [26], and is another diet aimed at CVD risk reduction. Rather than specific requirements, the DASH diet promotes dietary patterns of whole grains, vegetables, fruit, legumes, nuts, and seeds, while limiting the intake of full-fat dairy, red meats, sodium, and overly sugary items. Importantly, the DASH diet also emphasizes limiting sodium intake to less than 2300 mg daily to prevent the onset or worsening of hypertension [27]. As evidence has shown, hypertension is a strong risk factor for many causes of morbidity and mortality, notably cerebrovascular disease [27, 28].

Researchers evaluated the association between DASH diet adherence and all-cause and cause-specific mortality through a systematic review and meta-analysis [29]. Thirteen cohort studies, which include over 1.2 million patients, evaluated this relationship via a linear dose–response analysis and found that for every 5-point incremental increase in DASH diet adherence, the hazard ratio for all-cause mortality was reduced by 5%. The authors categorized the results and found a 4% decrease in CVD risk, a 3% decrease in stroke risk, and a 3% decrease in risk in cancer mortality. These results suggest an inverse relationship between better DASH diet adherence and lower mortality risks. The authors note the limitations related to accurately collecting dietary adherence data and variability regarding the hazard ratios amongst the different study populations, with those in the USA having higher mortality rates than counterparts from Asia or Europe [29].

In terms of these patterns and brain health, work is also underway to evaluate the impact of the Mediterranean-DASH Intervention for Neurodegenerative Delay (MIND) diet on cognition [30•, 31]. The MIND diet is akin to the Mediterranean and DASH (reviewed in the next section below) diets and features the consumption of vegetables, particularly, green leafy vegetables, berries, olive oil, nuts, whole grains, and low-fat sources of protein [32]. The MIND study is a 3-year, multicenter, RCT designed to evaluate the impact of the MIND diet on cognition in over 600 elderly individuals at risk for Alzheimer’s disease (AD). Outcome variables in this study include assessments of various cognitive domains and MRI-based metrics of neuropathology; this trial is now underway [30•].

### Plant-Based Diets

Plant-based diets represent another commonly discussed dietary pattern, which can be adapted for health, religious, or philosophical reasons. Several variations on plant-based dietary patterns exist, ranging from veganism (complete exclusion of animal-based foods) to moderate inclusion of meat, poultry, and fish [33]. Previous research has shown a possible association between reduced CVD risk and plant-based diets [34, 35]. More recent studies have adopted the plant-based dietary index (PDI) that allows for more standardized data comparison [36]. One systematic review and meta-analysis of prospective cohort studies aggregated results to determine the link between plant-based diet adherence and both CVD-related mortality and stroke outcomes [37]. With a total of 125 studies amounting to over 410,000 participants, the authors reported that greater adherence to a plant-based diet was correlated with significant reductions in cardiovascular mortality risk (pooled HR 0.92; 95% CI 0.86–0.99) and CVD incidence (pooled HR 0.90; 95% CI 0.82–0.98). They also found a non-significant reduction in total stroke incidence (pooled HR 0.86; 95% CI 0.69–1.08) [37].

Another study examined the effects of a vegetarian diet on participants who were omnivorous and overweight [38]. In this study, vegetarian was defined as ovo-lacto-vegetarian where diets included eggs and dairy, but did not include any meat, fish, or poultry. The authors compared the effects of low-calorie vegetarianism with the MD and performed a crossover after 3 months in 118 individuals. While both diets reduced the total calorie and fat intake, the vegetarian diet had significantly lower cholesterol intake and the MD had increased intake of protein intake. Both diets demonstrated statistically significant decreases in body weight, body mass index, and body fat content, without significant differences between the two. The vegetarian diet also showed a statistically significant decrease in low-density lipoprotein (LDL) cholesterol, while the MD evidenced greater triglyceride reduction. Study limitations included a predominantly female sample (78%), which stands in contrast to the TBI population largely comprised males. These results provide evidence of the benefits of both vegetarian and MD diets on total weight and body fat reduction, both of which contribute to CVD risk [38]. Similarly, one study looked at a low-fat vegan diet compared to the Mediterranean diet in 62 overweight adults and found greater reductions in body weight, lipid profile, and insulin sensitivity in the vegan diet cohort, versus a greater reduction in blood pressure with the Mediterranean diet [39]. The benefits of a plant-based diet (including CVD protection), likely arise from increased fiber, lower saturated, and unhealthy fats, and increased antioxidant and anti-inflammatory components of fruits and vegetables [34, 40•].

As mentioned above, there are studies on a plant-based diet and its impact on metabolic health, but there is limited research on its effect on cognition. Studies have found inconclusive evidence on the direct impact of a plant-based diet on cognitive health and aging [41, 42].

### Ketogenic Diet

A ketogenic diet consists of consuming foods that are high in fat, very low in carbohydrates, and moderate in protein content. This type of food intake deprives the body of glucose as the main source of energy and ketones are then employed. While the KD was introduced over a century ago, it was largely employed to treat epilepsy and thus enjoyed limited popular appeal [43]. As the possible applications of various forms of KDs have expanded, particularly for weight loss, the diet has seen an upsurge in interest over approximately the last 20 years.

Most studies evaluating the influence of a KD on metabolic health have taken place within specific patient cohorts, such as those diagnosed with obesity or DM-2, which can obscure the impact of a KD on specific metabolic factors outside of weight loss [44]. Moreover, given the specific patient cohorts included in these studies, the external validity of this literature can be somewhat limited. One narrative review studied the influence of a KD on blood pressure, and concluded that KDs can result in reduced blood pressure values, but do not induce significantly different changes relative to non-ketogenic diets and that the BP reduction is likely secondary to the associated weight loss [45]. Another systematic review and meta-analysis investigated the combined effect of exercise and low carbohydrate KD interventions on adiposity and metabolic health amongst individuals diagnosed as overweight and obese. They identified 7 studies and 278 individuals, with results demonstrating decreased triglycerides and waist circumference in those participating in KD combined with moderate to vigorous activity compared to usual diet with exercise over an average of 9 weeks. However, fasting glucose, total cholesterol, HDL, and LDL were not influenced [46].

With regard to neurologic conditions, review studies have concluded that while a KD has potential application for select diagnoses like multiple sclerosis and Alzheimer’s disease, there is relatively limited human trial data [47, 48]. Similar conclusions were reported with respect to brain injury. For instance, a scoping review reported that a KD had a positive impact on rodents with experimental TBI exposure [49]. However, there were few intervention studies on a KD in humans after TBI exposure, with extant trials working largely to establish feasibility and safety [50]. Of note, unlike other diets reviewed in this paper, a KD is not recommended for long-term use beyond approximately 6 months without medical supervision [51]. To this end, the utility of extended use to assist in the management of TBI recovery could be limited.

### Intermittent Fasting

Intermittent fasting (IF) is a method of dieting in which an individual undertakes a habitual pattern of eating their desired caloric intake within a set time frame. There are different modes of IF implementation, such as restricting calories to less than 500 cal every other day (alternate-day/AD), fasting 2 days a week while eating as per usual for the remainder of the week (5:2), and consuming a normal calorie limit within a short time-window each day (time-restricted eating /TRE) [52].

IF has many potential health benefits when compared to ad libitum diets. First, IF can be a more useful means of obtaining caloric restriction (CR) and weight loss than simple ad libitum CR [53]. IF can stabilize lipid and oxidative stress panels, correct blood-glucose concentrations, and increase insulin sensitivity [54–57]. Regarding brain health, a 3-year, longitudinal study correlated 5:2 IF to improved cognition while a TRE study extended it to geriatric adults with similar findings [58, 59]. This may be due to brain-derived neurotrophic factor (BDNF), a hormone that provides enhancements to cognition, neuroplasticity, and long-term potentiation within the hippocampus, and whose signaling is induced under the practice of both IF and physical exercise [60]. However, there are limited numbers of large studies or systematic reviews that determine the effects of IF in the human population, and therefore, this is an area for further research before recommending IF clinically, including amongst TBI survivors.

## Nutrition Education in Brain Injury Medicine

### Physiatrists Nutrition Knowledgebase and Counseling Practices

Historically, there has been a paucity of nutrition education within medical school curriculums [61], despite formal recommendations in 1985 by the US National Research Council Committee on Nutrition Education for all US medical schools to provide a minimum of 25 h of nutrition didactics as part of their medical curriculum [62]. Furthermore, a 2006 survey study of over 2300 medical students found that only 46% of 4th-year medical students believed nutrition education and counseling stills would be important or relevant for their future medical practice [63]. Specific to physiatrists, a recent pilot survey study amongst physiatry attendings, fellows, and residents found that 62% of respondents believed they did not have adequate nutrition knowledge to be able to effectively provide nutritional counseling to their patients, with over 85% of respondents believing additional nutrition education through web-based continuing medical education would enhance the clinical care they could provide to their patients [64].

### Evidence-Based Resources for Both Physiatrists and Brain Injury Survivors

Over the past decade, there has been a growing understanding amongst, clinicians, patients, and the lay public that modifiable cardiovascular risk factors, including evidence-based nutrition, are important for long-term health, and may be a primary driver of most chronic diseases. Thus, there are multiple web-based resources available for anyone with internet access. However, the difficulty is obtaining nutrition information that is grounded in the current best available balance of scientific evidence. To this end, in addition to the references contained within this narrative review, we have compiled a list of free, high-quality, evidence-based resources for both clinicians and patients to use for acquisition of nutrition knowledge (see Table 2) [65, 66, 67••, 68–70].

**Table 2:** Evidence-based resources for nutrition education

Organization	URL	Overview
Dietary Guidelines for Americans [65]	https://www.dietaryguidelines.gov	Official Dietary Guidelines for Americans from the US Government. Current edition 2020–2025
Canada’s Food Guide [66]	https://food-guide.canada.ca/en/	Official Dietary Guidelines from the Government of Canada. Contains an excellent infographic of an ideal plate of food
American College of Lifestyle Medicine [67••]	https://lifestylemedicine.org	Professional medical society that provides high-quality, evidence-based education to physicians and other medical-based specialists pertaining to lifestyle medicine interventions, including nutrition
NutritionCME.org [68]	https://www.nutritioncme.org	Website providing CME on the role of nutrition in long-term health, including brain health, without any commercial sponsorship. Many CME courses are free, and others are offered at low cost
The Nutrition Source [69]	https://www.hsph.harvard.edu/nutritionsource/	Official website from the Harvard University T.H. Chan School of Public Health, providing abundant evidence-based nutrition resources for both the lay public and clinicians
NutritionFacts.org [70]	https://nutritionfacts.org	Nonprofit organization providing evidence-based nutrition research through over 2000 short videos, along with an extensive blog archive

## Conclusions

While many individuals return to baseline after TBI exposure, a small proportion of individuals with moderate to severe TBI transition into a chronic recovery phase and suffer various effects of the injury and related disability. As physiatrists and brain injury medicine providers, as well as for the individuals with TBI exposure(s) themselves, professionals must be knowledgeable about the transition into the chronic recovery phase and advise individuals with TBI on identifying modifiable risk factors to improve their cardiovascular profile that can improve brain health. Most of the diets discussed share foods that are rich in fiber, vitamins, healthy fats, and phytochemicals, and low in saturated fats, added sugars, refined carbohydrates, and ultra-processed ingredients. Future research is needed to determine the effects of diet with TBI exposure, and specific symptoms that continue after injury, such as cognitive changes and fatigue. Nevertheless, there appears to be sufficient evidence for providers to encourage healthy eating since a reduction in metabolic dysfunction and CVD can be reasonably hypothesized to improve overall outcomes.

## Declarations

### Disclaimer

The contents of this work do not represent the views of the Department of the Veterans Affairs or the US Government. This is a US government work and not under copyright protection in the USA; foreign copyright protection may apply 2023.
